# Some Difficulties with V.A.D. Workers

**Published:** 1916-04-15

**Authors:** 


### THE EDITOR'S LETTER-BOX.

### Some Difficulties with V.A.D. Workers.

*To the Editor of* The Hospital.

Sir,—I have lately been put in charge of a small military hospital, for soldiers partially convalescent, in a provincial town. It is almost entirely staffed by V.A.D. workers, my only trained assistant being the night nurse. For keeping the house clean I have to depend on voluntary workers who have for the most part had no previous instruction, even in classes, and being women of the leisured class some of them learn for the first time the use of broom and house-flannel. I have no complaint to make of their zeal, and am often astonished at their quick and energetic method of tackling domestic problems. But I find it a great difficulty under these conditions to keep the floors quite as I should like. The boards are well laid, and stained, but within half an hour of being swept they look as dusty as before. Can any of your readers help to solve the difficulty? We have neither the money nor the labour required to get a good surface by means of beeswax and polish, but perhaps some matron who has struggled under similar circumstances to keep the wards smart may be able to offer a suggestion. I may add that not only do I seldom get the same workers for more than a week together, but I never know in the morning how many (if any) will appear. I am fortunate, however, in my kitchen staff, who are both efficient and regular in attendance.

Matron, Military Hospital.

